# The Role of ZIP9 and Androgen Receptor in the Establishment of Tight Junctions between Adult Rat Sertoli Cells

**DOI:** 10.3390/biology11050668

**Published:** 2022-04-26

**Authors:** Hassan Kabbesh, Ahmed Bulldan, Lutz Konrad, Georgios Scheiner-Bobis

**Affiliations:** 1 Institute for Veterinary Physiology and Biochemistry, School of Veterinary Medicine, Justus-Liebig-University Giessen, Frankfurter Str., D-35392 Giessen, Germany; hassan.kabbesh@vetmed.uni-giessen.de (H.K.); ahmed.bulldan@vetmed.uni-giessen.de (A.B.); 2 Center of Gynecology and Obstetrics, Faculty of Medicine, Justus-Liebig-University Giessen, Feulgenstr. 10-12, D-35392 Giessen, Germany; lutz.konrad@gyn.med.uni-giessen.de

**Keywords:** androgen receptor, Sertoli cells, testosterone, androgenic peptide, Erk1/2, CREB, claudin-1, ZO-1, JAM-3, ZIP9

## Abstract

**Simple Summary:**

Haploid male germ cells that originate at the onset of puberty from diploid precursor cells would be attacked by the innate immune system if they were not protected by the blood–testis barrier. This dynamic structure consists of tight junction proteins formed between epithelial Sertoli cells of the seminiferous tubules. Testosterone is the main regulator of this structure. We establish here, however, that testosterone actions affecting tight junctions, contrary to a decades-old assumption, are not mediated through the classical androgen receptor found in the cytosol or nuclei but through membrane-bound ZIP9, a multifunctional protein that is both a zinc transporter and androgen receptor. Our investigation establishes that ZIP9 plays a significant role in the regulation of tight junction protein expression and tight junction formation between Sertoli cells and that the involvement of ZIP9 should be considered in future studies dealing with the maintenance and protection of male fertility.

**Abstract:**

The blood–testis barrier (BTB) is formed from tight junctions (TJs) between Sertoli cells. This dynamic structure, which establishes an immune-privileged environment protecting haploid germ cells formed in puberty from cells of the innate immune system, protects male fertility. Testosterone produced in Leydig cells is one of the main regulators of TJ protein expression and BTB dynamics. Nevertheless, although it has been assumed that testosterone effects on TJs and BTB are mediated through the classical androgen receptor (AR), newer results call the importance of this receptor into question. ZIP9, a recently identified androgen receptor of plasma membranes, mediates testosterone effects that promote the expression of TJ proteins and TJ formation in a rat Sertoli cell line that lacks the classical AR. Although these findings suggest that ZIP9 mediates these testosterone effects, participation of the classical AR in these events cannot be excluded. Here we used immortalized adult rat Sertoli cells that express both ZIP9 and AR and addressed the involvement of these receptors in the stimulation of TJ protein expression and TJ formation in response to testosterone and to the androgenic peptide IAPG that acts via ZIP9. We find that both testosterone and IAPG trigger the so-called non-classical signaling pathway of testosterone and stimulate the expression of TJ-associated proteins and TJ formation. Silencing classical AR expression had no effect on the responses, whereas silencing of ZIP9 expression completely blocked them. Our results demonstrate that ZIP9 is the sole androgen receptor involved in the regulation of TJ protein expression and TJ formation at the BTB.

## 1. Introduction

Blood–tissue barriers exist between epithelial cells in order to control material exchange between blood and tissue but also to protect the tissue they surround from cells of the immune system. The blood–testis barrier (BTB) is a unique structure in mammalian male gonads that consists of tight junctions (TJs) formed between neighboring Sertoli cells [1]. By dividing the seminiferous epithelium into basal and adluminal compartments, the BTB fulfills two main functions: first, it controls the paracellular flow of water and nutrients across the Sertoli cell epithelia, and second, and maybe more important, it forms an immune-privileged environment [2,3]. This is necessary because haploid forms of male germ cells that first form in puberty are foreign to cells of the innate immune system and would trigger an immune response if they were not protected by the BTB [4,5,6]. The BTB is not a rigid structure but is rather dynamic and at times can disassemble to allow spermatogonia-derived diploid preleptotene spermatocytes to pass from the basal into the adluminal compartment where they further develop into diploid pachytene spermatocytes, haploid round spermatids, and finally haploid elongated spermatids, the actual germ cells [7,8]. 

Testosterone, a major factor for the establishment of male phenotype, behavior, and fertility, is produced in male gonads by somatic Leydig cells and acts on Sertoli cells to stimulate the expression of proteins that are either directly involved in the formation of TJs or are associated with them. Thus, testosterone is absolutely essential for the formation and maintenance of the BTB [2,4,9,10]. 

The testosterone receptor responsible for the stimulation of the expression of TJ proteins and the formation of the BTB, however, remains to be defined. The classical androgen receptor (AR) has for decades been undisputed as the mediator of all testosterone effects, including the stimulation of TJ formation at the BTB; however, recent investigations have challenged this assumption [11,12,13]. ZIP9, a rather recently identified membrane-bound androgen receptor and zinc transporter from the family of ZRT/IRT-like proteins (ZRT = zinc-regulated transporter; IRT = iron-regulated transporter) [14,15], mediates testosterone-induced signaling events in the rat-derived Sertoli cell line 93RS2 that lead to increased expression of claudin-1 and claudin-5 and to enhanced TJ formation [13]. Nevertheless, as this particular cell line does not express the classical AR but only ZIP9 [16], it cannot be excluded that the classical AR might also be involved in the physiological stimulation of TJ formation.

The current investigation, using a recently established cell line of immortalized adult rat Sertoli cells that express both ZIP9 and the classical AR [17], addresses the effects of testosterone after silencing the expression of ZIP9 and compares them with the effects of a testosterone-mimetic tetrapeptide designed to specifically fit within the androgen binding site of ZIP9 [18].

## 2. Materials and Methods

### 2.1. Primary Adult Rat Sertoli Cells 

Primary adult rat Sertoli cells were isolated using enzymatic digestion and were treated by conditional reprogramming to generate the adult rat Sertoli cell line PASC1, as described [17]. Briefly, the tubules from adult rat testis were exposed to 2 steps of enzymatic digestion. The mixture obtained was then layered on Percoll gradients after washing with PBS, the supernatants were discarded, and the remaining tubules were subjected to a third digestion. The mixture was filtered through cell strainers to get SC clusters which were cultured with conditioned medium (CM) supplemented with 10 µM ROCK inhibitor Y-27632. Hypotonic shock was performed at day 3 and SC characterization can be undertaken starting from day 5 [17].

### 2.2. Measurement of Transepithelial Resistance (TER)

TER measurements were performed as described [17]. Briefly, 6.0 × 10^4^ cells/cm^2^ of androgen receptor (AR)- or ZIP9-knockdown PASC1 or non-treated PASC1 cells were seeded on 0.4 μm inserts of 24-well plates (Greiner bio one, Frickenhausen, Germany) and cultured for 48 h until they reached confluence. Testosterone (10 nM; Sigma-Aldrich, Steinheim, Germany) or synthetic peptide-1 (1 µM; P1) were then added to the inserts. As testosterone was dissolved in ethanol (vehicle), the same volume of ethanol was added to the peptide-treated cells and untreated controls. TER measurements were made with a Millicell ERS-2 epithelial Volt-Ohm meter (Merck Millipore, Darmstadt, Germany). ΩXcm^2^ was calculated according to the protocol of the manufacturer by setting the resistance of cell-free inserts to zero.

### 2.3. Plasma Membrane Labeling with Testosterone-BSA-FITC 

PASC1 cells were cultured as described above in 24-well plates at a density of 3 × 10^3^ cells/well until reaching a confluence of approximately 80%. The cells were then treated with either 10 nM testosterone or 1 µM of P1 for 1 h. Controls received only the testosterone vehicle (ethanol). Thereafter, testosterone 3-(O-carboxymethyl)-oxime:bovine serum albumin-fluorescein isothiocyanate conjugate (T-BSA-FITC; Sigma-Aldrich, Steinheim, Germany) dissolved in Tris buffer (pH 7.2) was added to each well at a final concentration of 10 µM, and incubation was continued at room temperature for another 20 min. The medium was then removed by aspiration. In order to label nuclei, cells were fixed at room temperature with 3.7% formaldehyde that contained 20 ng of 4,6-diamino-2-phenylindole (DAPI). After 15 min, the formaldehyde/DAPI solution was removed by aspiration. Cells were washed with Dulbecco’s phosphate-buffered saline (DPBS, Gibco, Schwerte, Germany) and then overlayed with 400 µL PBS before imaging. Images were taken by an inverse Olympus IX81 microscope (Olympus, Hamburg, Germany). Non-specific binding was assessed by incubating the cells with 10 µM BSA-FITC (lacking testosterone) dissolved in Tris buffer (pH 7.2) for 20 min at room temperature. 

### 2.4. Preparation of Cell Lysates from PASC1

A total of 3 × 10^4^ PASC1 cells/dish were grown in 5 cm culture dishes as described above. Cells were then incubated for 24 h with 1% FCS before testosterone or P1 were added to the medium to reach the desired final concentration. The ethanol vehicle for testosterone was added to the peptide-treated cells and untreated controls at the same concentration as in cell cultures treated with testosterone. After 24 h, the medium was aspired and the cells were washed twice in ice-cold DPBS. Cells were then incubated with 400 µL lysis buffer (Cell Signaling Technologies, Frankfurt, Germany) containing 1 µM PMSF, 1× protease inhibitor cocktail (Roche, Mannheim, Germany), and 2 µg/mL pepstatin that was added immediately before use. All further steps were carried out on ice. After 5 min of incubation, cells were detached using a cell scraper. The suspensions were then transferred into 1.5 mL vials and sonicated 5 times for 1 s each with intervals of 1 s. After centrifugation of the lysates at 4 °C and 13,000× *g* for 10 min, the protein concentration in the supernatants was determined at 540 nm using a bicinchoninic acid protein assay reagent kit (Pierce, Southfield, MI, USA) and a Labsystems (Helsinki, Finland) plate reader. The BSA protein standard contained lysis buffer at the same concentration as in the samples from the cell lysates. Supernatants were then aliquoted and maintained at −20 °C until further use.

### 2.5. Western Blotting

A total of 10 µg protein from PASC1 cell lysates was run on SDS-PAGE gels containing 10% acrylamide and 0.3% N,N′-methylene-bis-acrylamide. Biotinylated proteins (Cell Signaling Technologies) were run in parallel as molecular weight markers. After electrophoresis, proteins were semi-dry electro-blotted onto PVDF membranes (Merck Chemicals GmbH, Schwalbach, Germany) for 30 min at 0.5 V/cm^2^. The membranes were then incubated for 1 h at room temperature in 5% (*w*/*w*) non-fat dry milk. Primary antibodies against phospho-Erk1/2, total-Erk1/2, or beta-actin were diluted according to the recommendations of the manufacturers and then incubated with the PVDF membranes overnight at 4 °C (see Supplementary Table S1 for all antibodies with dilutions and manufacturers). After washing in DPBS, the membranes were incubated under continuous mild agitation on an orbital shaker for 60 min at room temperature with a horseradish peroxidase-conjugated secondary antibody. An anti-biotin, HRP-conjugated antibody at a dilution of 1:2000 was also included in the mixture containing the secondary antibody in order to detect the biotinylated molecular weight marker. Antibody-labeled protein bands were visualized by enhanced chemiluminescence [19]. 

### 2.6. Immunofluorescence

PASC1 cells were plated at a density of 3.0 × 10^4^ on 24-well plates (1 mL/well) and incubated 48 h at 37 °C and 5% CO_2_ until a confluence of roughly 80% was reached. Cells were rinsed with DPBS and fixed with 100% ice-cold methanol (Roth, Karlsruhe, Germany) on ice for 10 min directly or after stimulation with vehicle, 10 nM testosterone, or 1 µM P1 for 24 h. Cells were then washed with DPBS three times for 5 min, and then incubated in blocking solution (PBS with 3% BSA and 0.3% Triton X-100, both from Sigma-Aldrich) for 1 h at room temperature. The blocking solution was replaced by fresh blocking solution containing the primary antibodies for detection of either AR, p-Erk1/2, p-CREB/p-ATF-1, claudin-1 (Cldn-1), or zonula occludens-1 (ZO-1) (Supplementary Table S1) and incubated overnight at 4 °C. Cells were washed 3 times with DPBS for 3 min each at RT on an orbital shaker, freshly made blocking solution with the appropriate Alexafluor 488-conjugated donkey anti-rabbit secondary antibody was added for 1 h at room temperature on an orbital shaker. After washing 3 times with DPBS, images were obtained using an inverse Olympus IX81 microscope equipped with a fluorescence system. A total of 30 cells from 3 independent experiments (30 × 3) localized closest to the diagonals of the square optical field were selected and the manually surrounded green fluorescence was quantified by ImageJ (available at http://rsbweb.nih.gov/ij/, last accessed 28 February 2022) following the protocol of the software provider. The data obtained were analyzed by GraphPad Prism5 (GraphPad Software, Inc., La Jolla, CA, USA).

### 2.7. RNA Extraction and Quantitative Real Time PCR 

Primers were designed with http://www.ncbi.nlm.nih.gov/tools/primer-blast (Accessed on 28 February 2022) and were all intron-spanning. Total RNA was isolated from PASC1 with the RNeasy Mini-Kit (Qiagen, Hilden, Germany) and subjected to DNase I treatment as described by the manufacturer. The Reverse Transcription-System first strand cDNA synthesis kit (Invitrogen, Darmstadt, Germany) was used for cDNA synthesis, as recommended. Quantitative real-time PCR (qRT-PCR) amplification was performed in duplicate with iQ TM SYBR Green Super-mix (Bio-Rad; Munich; Germany) on the iCycler iQ System (Bio-Rad). After an initial heating at 94 °C for 5 min, 40 amplification cycles were performed consisting of an initial denaturation of the DNA double strands at 94 °C for 14 s, an annealing phase of the primers at 59 °C for 30 s, and extension of the primers for 15 s at 72 °C. A finalizing extension at 72 °C was carried out for 10 min. Gene expression was measured after reaching the ct value and calculated using the Delta–Delta Ct method [20]. Detection of GAPDH-specific mRNA/cDNA served as normalization of the quantification. Oligonucleotides used in the qRT-PCR, their annealing position, and the expected size of the amplificates are listed in Supplementary Table S2.

### 2.8. Silencing Expression of ZIP9 or AR via siRNA

For silencing ZIP9 or AR expression, PASC1 cells were treated with commercially available siRNA directed against the expression of ZIP9 or the AR by following the manufacturer’s protocol (Silencer ^®^ Select, siRNA Invitrogen, Darmstadt, Germany). The following oligonucleotides were used: 5′ GGAUUAAGUAAGAGCAGUAtt3′ and 5′ UACUGCUCUUACUUAAUCCta 3′ (ZIP9-siRNA for ZIP9 silencing) and 5′CCGGAAAUGUUAUGAAGCAtt3′ plus 5′ UGCUUCAUAACAUUUCCGGag 3′ (AR-siRNA for AR silencing). Negative control siRNA (nc-siRNA) was provided in the siRNA kit from the same manufacturer. After incubation of PASC1 for 48 h with ZIP9-siRNA, AR-siRNA, or nc-siRNA, preparation of samples for qRT-PCR, TER measurement, or immunofluorescence experiments were carried out as described above.

### 2.9. Statistical Analysis

All experiments were repeated independently at least three times with duplicate samples. Means and standard error (SEM) values from all experiments were used for analysis. Comparison of the means between more than two groups was made by one-way analysis of variance (ANOVA) followed by Dunnett multiple comparison test using GraphPad Prism software (Version 5.0, GraphPad Inc., La Jolla, CA, USA). Significance was accepted at *p* < 0.05. 

## 3. Results

### 3.1. Silencing AR or ZIP9 Expression by siRNA

**Figure 1:**
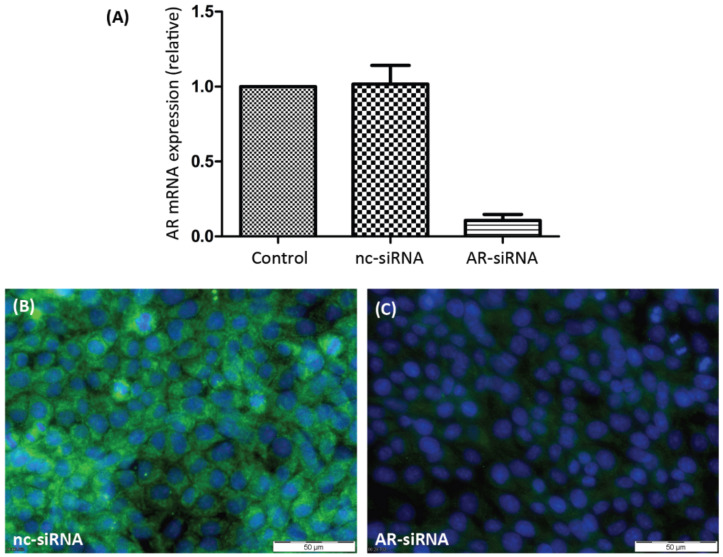
Detection of AR mRNA or protein in PASC1 cells. Cells were treated with either nc-siRNA or AR-siRNA. (**A**) qRT-PCR showing expression of AR-specific mRNA/cDNA. (**B**) Detection of AR protein by immunofluorescence before and (**C**) after silencing AR-specific mRNA expression. Green fluorescence corresponds to AR and nuclei are stained blue.

**Figure 2:**
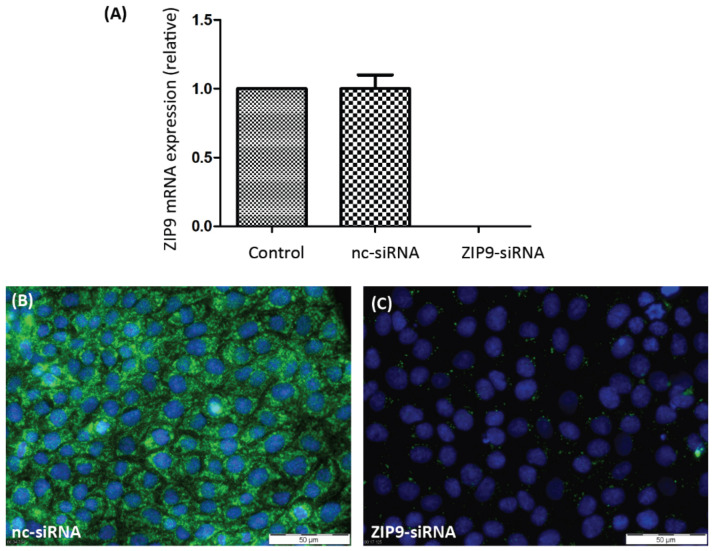
Detection of ZIP9 mRNA or protein in PASC1 cells. Cells were treated with either nc-siRNA or ZIP9-siRNA. (**A**) qRT-PCR showing expression of ZIP9-specific mRNA/cDNA. (**B**) Detection of ZIP9 protein by immunofluorescence before and (**C**) after silencing ZIP9-specific mRNA expression. Green fluorescence corresponds to ZIP9 and nuclei are stained blue.

In order to evaluate the participation of either of the two androgen receptors in PASC1 cell responses in the following experiments, it was necessary to selectively silence their expression. As Figure 1A shows, the expression of AR-specific mRNA was greatly suppressed by AR-siRNA. As a consequence, the expression of AR protein (Figure 1C) was also prevented by the AR-siRNA. ZIP9-directed siRNA was similarly successful in preventing expression of ZIP9 mRNA (Figure 2A) or protein (Figure 2B,C).

### 3.2. Responsiveness of PASC1 Cell AR towards Testosterone or the ZIP9-Targeting Peptide IAPG

The classical AR is a transcription factor that responds to testosterone or dihydrotestosterone through dimerization and translocation of the dimers from the cytosol into the nucleus, where they can regulate gene expression [21,22]. In order to ensure that the AR of PASC1 cells is responsive to testosterone and does not interact with the ZIP9-targeting IAPG peptide, its ability to translocate into the nucleus in response to testosterone or IAPG exposure was addressed.

**Figure 3:**
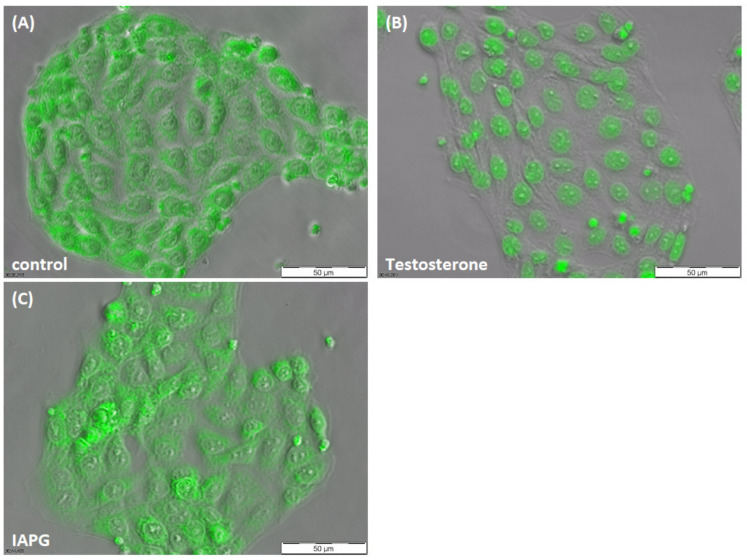
Immunofluorescence of AR in PASC1 cells. Cells were treated with testosterone or IAPG for 2 h. (**A**) AR (green) was distributed in the cytoplasm and in the nucleus in the untreated control. (**B**) After exposure to 10 nM testosterone, AR translocated into the nuclei. (**C**) There was no change in AR localization with 10 µM IAPG.

As Figure 3A shows, green fluorescence corresponding to AR in unstimulated PASC1 cells was present in the cytosol and also partially within the nuclei. After exposure to testosterone, all green fluorescence was found within the nuclei, demonstrating that the AR of PASC1 is responsive to the androgen (Figure 3B). A translocation of the AR from the cytosol into the nucleus did not occur upon exposure to IAPG (Figure 3C), although the peptide was used at a 1000-fold higher concentration (10 µM) than testosterone (10 nM). 

### 3.3. Binding of the IAPG Peptide or Testosterone to the Androgen Binding Site of ZIP9

Testosterone-BSA-FITC (T-BSA-FITC) is a fluorescent, plasma membrane-impermeable testosterone analogue that can be used to detect membrane-bound testosterone receptors with an extracellular androgen binding site [16,23,24]. It is suitable to label the androgen binding site ZIP9 of the Sertoli cell line 93RS2 and also of L6 myoblasts [13,18]. Its binding can be prevented by either testosterone or by specific tetrapeptides such as IAPG modeled to fit within the ZIP9 androgen binding site [13,18]. 

**Figure 4:**
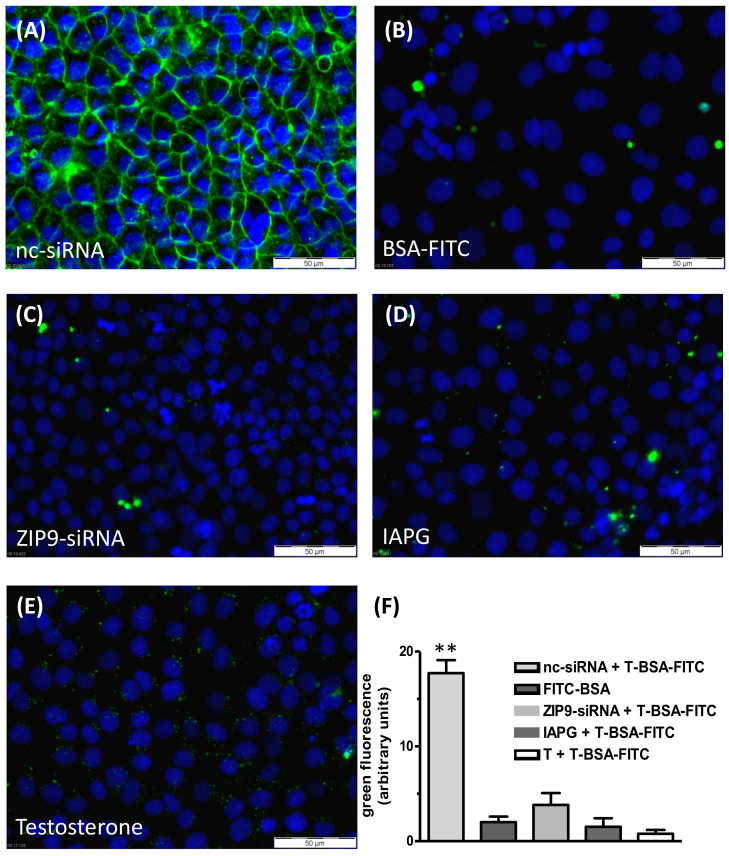
Testosterone-BSA-FITC (T-BSA-FITC) membrane labeling of PASC1 cells. T-BSA-FITC or BSA-FITC are visualized as green fluorescence and nuclei are stained blue. (**A**) T-BSA-FITC labeled membranes of cells treated with nc-RNA. (**B**) BSA-FITC lacking the testosterone moiety did not stain the membranes. (**C**) There was also no staining after ZIP9 knockdown. (**D**) IAPG (10 µM) or (**E**) testosterone (10 nM) prevented membrane labeling by T-BSA-FITC. (**F**) Statistical analysis of the green fluorescence (*n* = 28; means ± SEM; ** *p* ≤ 0.01).

T-BSA-FITC was shown to label the surface of PASC1 cells (Figure 4A). The labeling was due to the binding of the testosterone moiety of T-BSA-FITC, since BSA-FITC that lacks the testosterone component failed to label the membrane structure (Figure 4B). These two experiments indicate the presence of an extracellularly accessible androgen binding site. This site consists of ZIP9, since suppression of its expression by siRNA prevented the binding of T-BSA-FITC (Figure 4C). As expected from the earlier experiments mentioned above, both IAPG and testosterone specifically interact with membrane-bound ZIP9 of PASC1 cells, as they displaced T-BSA-FITC from its androgen binding site (Figure 4D,E). The statistical analysis of the green fluorescence is summarized in Figure 4F.

### 3.4. Stimulation of Erk1/2 Phosphorylation by Testosterone or IAPG

Activation of Erk1/2 by phosphorylation occurs in the so-called non-classical signaling pathway of testosterone [25,26]. In the Sertoli cell line 93RS2 that lacks the classical AR, testosterone stimulated the phosphorylation of Erk1/2 with an EC_50_ of 0.411 ± 1.54 nM [13].

The IAPG peptide stimulated phosphorylation of the same kinase with an EC_50_ of 1.39 ± 1.36 µM. Maximum Erk1/2 phosphorylation was achieved by 10 nM testosterone or by 10 µM IAPG [13].

**Figure 5:**
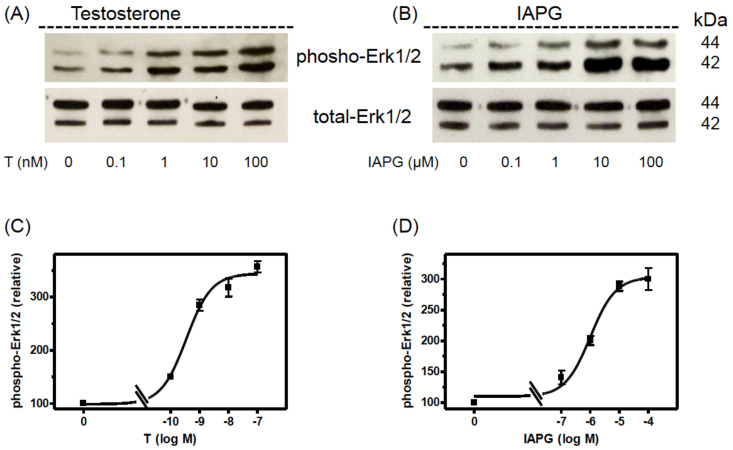
Western blot analysis of phospho-Erk1/2. PASC1 cells were stimulated for 24 h with various concentrations of testosterone or IAPG. (**A**) Phospho-Erk1/2 detection after stimulation with testosterone and (**B**) after stimulation with IAPG. In (**A**,**B**) the total amount of Erk1/2 was not affected. (**C**) Semi-logarithmic plot of relative p-Erk1/2 signals revealed for testosterone an EC_50_ of 0.35 ± 1.29 nM and (**D**) for IAPG an EC_50_ of 1.02 ± 1.36 µM. For each diagram: *n* = 3; means ± SEM. Original Western blots are shown in Supplementary Figure S1.

It was therefore of interest to address testosterone and IAPG effects on Erk1/2 in the adult Sertoli PASC1 cell line and to compare them with those in the rat Sertoli cell line 93RS2 [13]. As the Western blots in Figure 5 show, testosterone or IAPG did not affect total expression of Erk1/2 or beta-actin, but they stimulated within 30 min Erk1/2 phosphorylation in a concentration-dependent manner. The EC_50_ values were determined to be 0.35 ± 1.29 nM for testosterone and 1.02 ± 1.36 µM for IAPG (Figure 5C,D), which are in very good agreement with the EC_50_ values obtained for Erk1/2 phosphorylation in the rat Sertoli cell line 93RS2 [13]. The same also applies for the concentrations needed for maximum stimulation of Erk1/2 phosphorylation which also here was achieved by either 10 nM testosterone or 10 µM IAPG (Figure 5) [13]. Based on these results, all further experiments were carried out using these concentrations of testosterone or IAPG. 

### 3.5. Identification of the Androgen Receptor Involved in Erk1/2 Phosphorylation 

**Figure 6:**
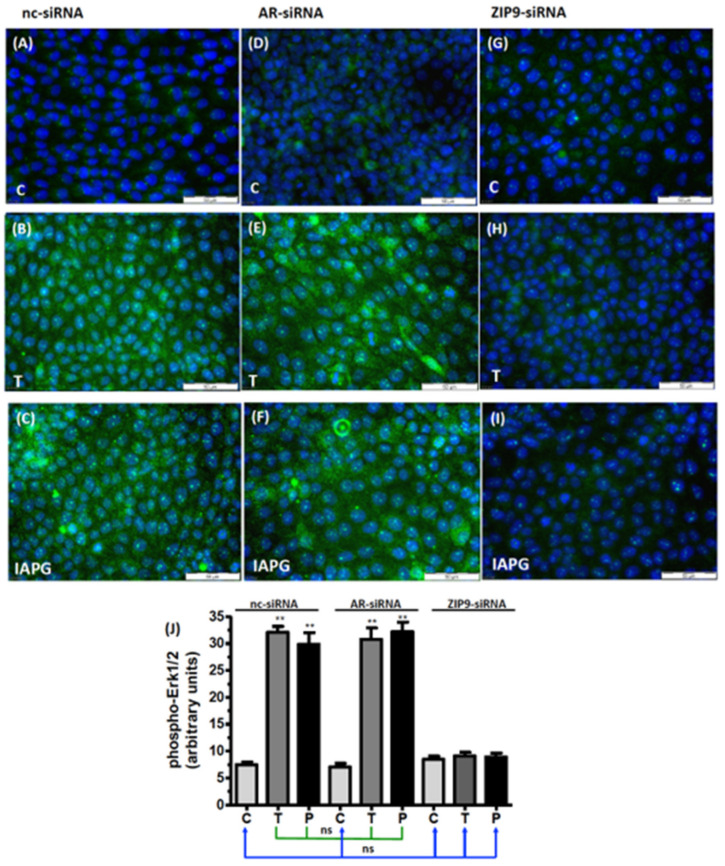
Immunofluorescence of phospho-Erk1/2 in PASC1 cells. Incubation with vehicle (C), 10 nM testosterone (T), or 10 µM IAPG (P) was carried out for 24 h. Nuclei are stained blue, and phospho-Erk1/2 is represented by green fluorescence. (**A**–**C**) Phospho-Erk1/2 in cells treated with nc-siRNA; (**D**–**F**) phospho-Erk1/2 in cells treated with AR-siRNA; (**G**–**I**) phospho-Erk1/2 in cells treated with ZIP9-siRNA. (**J**) Statistical analysis of green fluorescence shown in panels A–I (*n* = 3 × 25; means ± SEM; ** *p* ≤ 0.01; ns = not significant).

A possible involvement of either AR or ZIP9 in the stimulation of Erk1/2 was addressed by assessing immunofluorescence in PASC1 cells after selectively silencing AR or ZIP9 expression by AR-siRNA or ZIP9-siRNA. In cells that were exposed to nc-siRNA only, which served as a control, green fluorescence reflecting the presence of phosphorylated Erk1/2 was low (Figure 6A) relative to the signal obtained in cells exposed to testosterone (Figure 6B) or IAPG (Figure 6C). Silencing AR expression yielded similar results: untreated control cells (Figure 6D) displayed a low level of green fluorescence, whereas cells exposed to testosterone (Figure 6E) or IAPG (Figure 6F) responded with augmented phosphorylation of Erk1/2. The stimulatory effects of testosterone or IAPG on Erk1/2 phosphorylation in the presence of either nc-siRNA or AR-siRNA were not significantly different from each other (Figure 6J). 

Silencing of ZIP9 expression entirely prevented stimulation of Erk1/2 phosphorylation by either testosterone or IAPG. In the absence of the two substances (Figure 6G), green fluorescence corresponding to p-Erk1/2 was present at the low, basal level observed in the other control incubations (Figure 6A,D) and remained at that level regardless of treatment with either testosterone (Figure 6H) or IAPG (Figure 6I). Results obtained after silencing the expression of ZIP9 were not significantly different from those obtained under control conditions with nc-siRNA or AR-siRNA (Figure 6J).

### 3.6. Involvement of AR or ZIP9 in the Stimulation of CREB/ATF-1 Phosphorylation by Testosterone or IAPG

**Figure 7:**
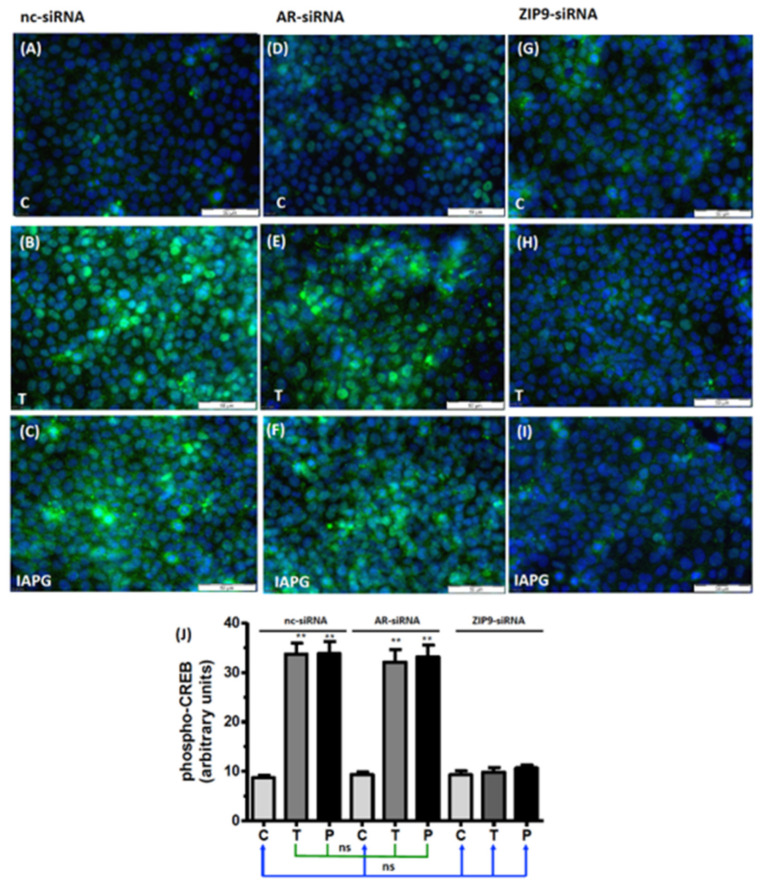
Immunofluorescence of p-CREB/p-ATF-1. Incubation with vehicle (C), 10 nM testosterone (T), or 10 µM IAPG (P) was carried out for 24 h. Nuclei are stained blue and p-CREB/ATF-1 is green. (**A**–**C**) p-CREB/p-ATF-1 in cells treated with nc-siRNA. (**D**–**F**) p-CREB/p-ATF-1 in cells treated with AR-siRNA. (**G**–**I**) p-CREB/p-ATF-1 in cells treated with ZIP9-siRNA. (**J**) Statistical analysis of green fluorescence (*n* = 3 × 25; means ± SEM; ** *p* ≤ 0.01; ns = not significant).

Phosphorylation of the transcription factors CREB and ATF-1 constitutes a downstream event of the non-classical signaling pathway of testosterone [12,13,27]. Because of the similarity of the phosphorylation domains of CREB and ATF-1, the antibody commonly used to detect them does not distinguish between them [13,28]. In cells treated with nc-siRNA, both testosterone and IAPG stimulated CREB/ATF-1 phosphorylation, which was visible as green fluorescence within the nuclei (Figure 7A–C). Cells treated with AR-siRNA remained responsive to testosterone- or IAPG-induced phosphorylation of CREB/ATF-1 (Figure 7D–F) to the same extent as cells treated with nc-siRNA (Figure 7J). Testosterone and IAPG failed to stimulate phosphorylation of CREB/ATF-1 in cells treated with ZIP9-siRNA (Figure 7G–I). The results shown here did not differ from the results obtained after nc-siRNA or AR-siRNA treatment of cells under control conditions (Figure 7J).

### 3.7. Involvement of AR or ZIP9 in the Stimulation of ZO-1 Expression by Testosterone or IAPG 

**Figure 8:**
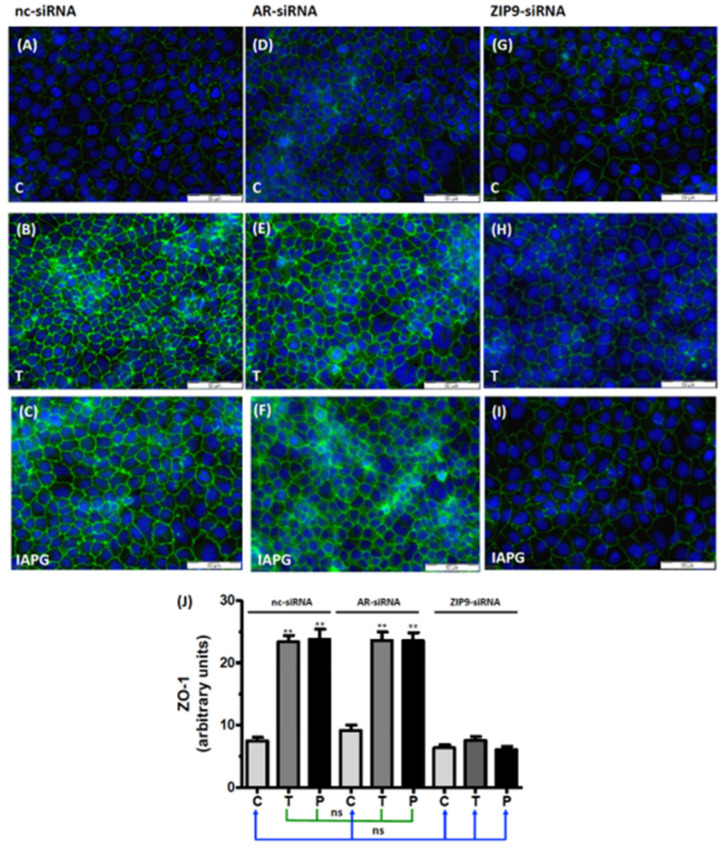
Immunofluorescence of ZO-1 expression in PASC1. Cells were treated with vehicle (C), 10 nM testosterone (T), or 10 µM IAPG (P) for 24 h. Nuclei are shown as blue and ZO-1 is green. (**A**–**C**) ZO-1 in cells treated with nc-siRNA. (**D**–**F**) ZO-1 in cells treated with AR-siRNA. (**G**–**I**) ZO-1 in cells treated with ZIP9-siRNA. (**J**) Statistical analysis of green fluorescence shown in panels A–I (*n* = 3 × 25; means ± SEM; ** *p* ≤ 0.01; ns = not significant).

ZO-1 is a cytosolic multi-domain protein that interacts with the TJ-forming proteins occludin, junctional adhesion molecules (JAM), or claudins and links these via actin to the cytoskeleton [29,30,31,32,33]. Thus, ZO-1 is found on the cytosolic surface of the plasma membrane. Untreated PASC1 cells that had been incubated with nc-siRNA showed modest membrane-associated green fluorescence corresponding to ZO-1 (Figure 8A). In cells exposed to testosterone (Figure 8B) or to IAPG (Figure 8C), the ZO-1-corresponding signal was significantly intensified (Figure 8J), indicating that both compounds stimulated its expression. Similar effects were observed when the cells were treated with AR-siRNA prior to their exposure to either testosterone or IAPG (Figure 8D–F). The ZO-1 fluorescence was unchanged by the ligands; however, when ZIP9 expression had been prevented by ZIP9-siRNA (Figure 8G–I). The levels of ZO-1 fluorescence stimulated by testosterone and IAPG in cells that had been treated with either nc-siRNA or AR-siRNA are not significantly different (Figure 8J). The same applies for control cells treated with nc-siRNA or AR-siRNA and all ZIP9-siRNA-treated cells (Figure 8J).

### 3.8. Participation of AR or ZIP9 in the Stimulation of Cldn-1 and JAM-3 Expression by Testosterone or IAPG

**Figure 9:**
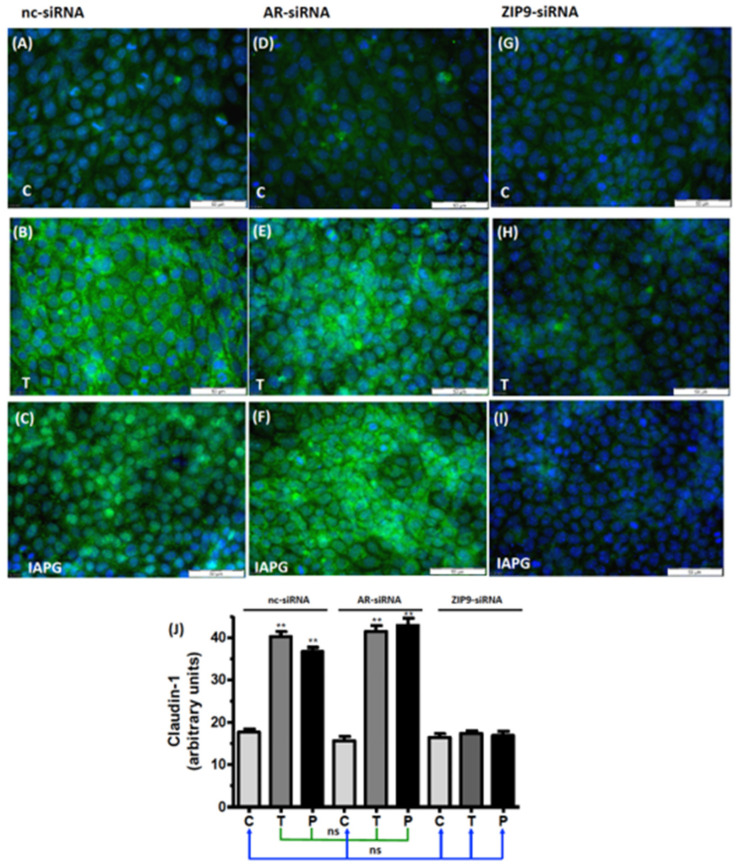
Detection of Cldn-1 by immunofluorescence. Incubation with vehicle (C), 10 nM testosterone (T), or 10 µM IAPG (P) was carried out for 24 h. Nuclei are shown as blue and Cldn-1 as green. (**A**–**C**) Cldn-1 in cells treated with nc-siRNA. (**D**–**F**) Cldn-1 in cells treated with AR-siRNA. (**G**–**I**) Cldn-1 in cells treated with ZIP9-siRNA. (J) Statistical analysis of green fluorescence shown in panels A-I (*n* = 3 × 25; means ± SEM; ** *p* ≤ 0.01; ns = not significant).

The expression of Cldn-1 and JAM-3 in the seminiferous tubule epithelia is regulated by testosterone [12,13,34,35,36]. Both proteins interact with ZO-1 and contribute to the formation of TJ and the tightening of the BTB. Testosterone and IAPG stimulated the expression of Cldn-1 in cells that had been treated with either nc-siRNA (Figure 9A–C) or AR-siRNA (Figure 9D–F) but failed to do so in cells where ZIP9 expression had been knocked down by ZIP9-siRNA (Figure 9G–I).

**Figure 10:**
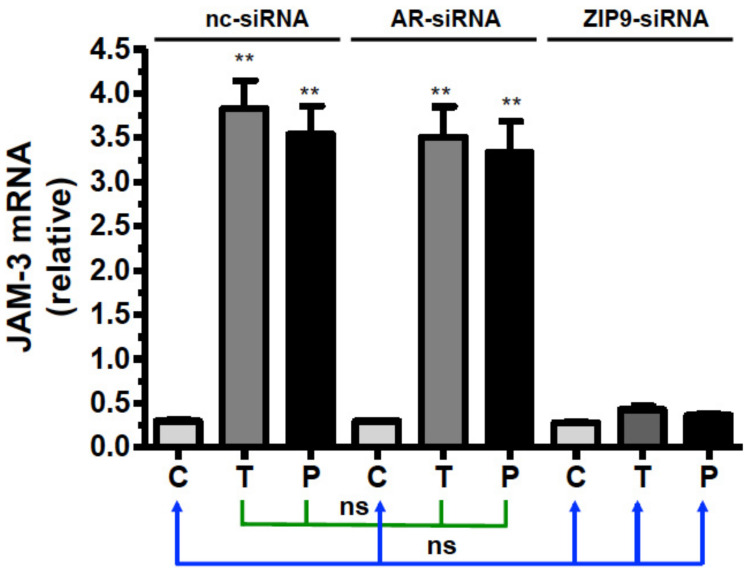
Detection of JAM-3-specific mRNA by qRT-PCR. PASC1 cells were treated with nc-siRNA, AR-siRNA, or ZIP9-siRNA and afterwards incubated for 24 h with vehicle (C), 10 nM testosterone (T), or 10 µM IAPG (P). For each data point: *n* = 3 × 2; means ± SEM; ** *p* ≤ 0.01; ns = not significant.

Basal JAM-3 mRNA expression in PASC1 cells treated with nc-siRNA, AR-siRNA, or ZIP9-siRNA (Figure 10) was upregulated by testosterone and IAPG only in cells that had been treated with nc-siRNA or AR-siRNA (Figure 10). In cells that had been incubated with ZIP9-siRNA, JAM-3 mRNA expression remained at basal levels after agonist treatment, like that observed in controls (Figure 10). 

### 3.9. Involvement of AR or ZIP9 in Testosterone- or IAPG-Induced TJ Formation

**Figure 11:**
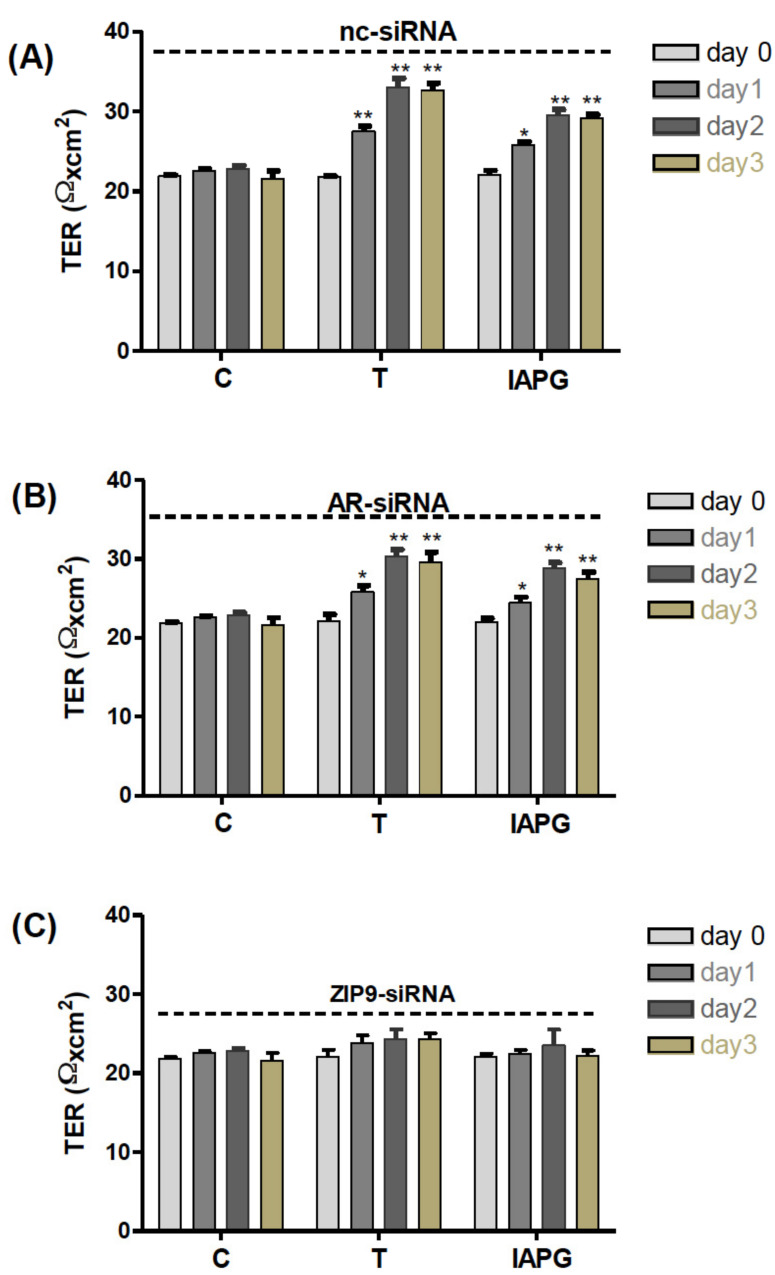
Transepithelial electrical resistance (TER) across adult Sertoli cell layers. The PASC1 cells were treated with either nc-siRNA (**A**), AR-siRNA (**B**), or ZIP9-siRNA (**C**) before being exposed to vehicle (C), 10 nM testosterone (T), or 10 µM or IAPG (P). Measurements of TER were taken after 0, 24, 48, and 72 h. For each data point: *n* = 6; means ± SEM; * *p* ≤ 0.05; ** *p* ≤ 0.01.

All the above results point towards the significant involvement of ZIP9 in the formation of TJ between Sertoli cells. TER measurements conducted over several days were consistent with this conclusion. Testosterone or IAPG stimulated PASC1 cells that had been treated with nc-siRNA to form tighter TJ than the control already after 1 day of treatment, as demonstrated by increased TER (Figure 11A). Testosterone and IAPG had the same effect on cells that had been treated with AR-siRNA to knock down AR expression (Figure 11B). When ZIP9 expression was knocked down by ZIP9-siRNA, testosterone and IAPG failed to stimulate TJ formation between the PASC1 cells (Figure 11C).

## 4. Discussion

The current investigation addressed the involvement of the classical AR or of ZIP9 in the non-classical signaling pathway of testosterone and in the stimulation of TJ protein expression and TJ formation. These experiments employed adult rat Sertoli cells that can be maintained at length in culture without losing their Sertoli cell characteristics [17]. These PASC1 cells express both the classical AR and ZIP9. Testosterone and a tetrapeptide designed to fit the androgen binding site of ZIP9, IAPG, were used as stimulants. This peptide was shown previously to induce ZIP9-mediated androgenic effects [13,18].

In a first step, the ability of ZIP9 to recognize not only testosterone but also the IAPG peptide was addressed. Each of the two molecules prevented labeling of the PASC1 membrane surface by T-BSA-FITC, a testosterone analogue that cannot penetrate the plasma membrane. Labeling of the plasma membrane by T-BSA-FITC also failed when ZIP9 expression was prevented by siRNA, indicating that ZIP9 is the sole androgen receptor in PASC1 cell membranes with an extracellularly accessible androgen binding site. 

The classical AR is an androgen-activated transcription factor that is localized in the cytosol or nuclei. When stimulated by testosterone or dihydrotestosterone, cytosolic AR form dimers that move into the nuclei to regulate gene expression [21,22]. The present study showed that the AR of PASC1 cells responds to testosterone accordingly; it moves from the cytosol to the nucleus, thus demonstrating that PASC1 cells express a functioning AR. In contrast, IAPG fails to induce a similar translocation of the AR, consistent with the peptide being highly specific towards the androgen binding site of ZIP9 and not interacting with the AR. These two experiments suggest that testosterone binds to the androgen binding site of both AR and ZIP9, whereas the peptide IAPG only interacts with the androgen binding site of ZIP9. 

Testosterone and IAPG stimulate in a concentration-dependent manner Erk1/2 phosphorylation in PASC1 cells, testosterone with an EC_50_ of 0.35 ± 1.29 nM, and IAPG with an EC_50_ of 1.02 ± 1.36 µM, as determined in Western blots. These values are within the same range as those obtained under similar conditions in the 93RS2 Sertoli cell line [13]. Maximal effects were obtained in both cases at 10 nM testosterone or 10 µM IAPG. 

Activation of Erk1/2 in Sertoli cells is part of the Src/c-Raf/Erk1/2/CREB(ATF-1) signaling module that promotes the expression of TJ proteins and the formation of TJ. Testosterone and IAPG were found to stimulate phosphorylation of Erk1/2 and CREB/ATF-1 only in the presence of ZIP9, whereas the presence of AR is not important for the phosphorylation status of these proteins. 

Similar observations were made with respect to the stimulation of the expression of TJ-associated ZO-1 and TJ-forming Cldn-1 or JAM-3. Thus, the presence of ZIP9 was essential for the stimulation of the expression of the three TJ-associated proteins addressed in this investigation. These results show that TJ formation fully depends on the presence of ZIP9 and is completely independent of the presence of the AR. 

ZO-1, claudins, and JAM-3 are essential for the formation and maintenance of the BTB. Ensuring the dual functionality of this structure as a physical and immunological barrier is an absolute presupposition for the protection of male fertility [1,37,38], and BTB defects that weaken its integrity are associated with impaired spermatogenesis and testicular dysgenesis syndrome; malfunctioning of the BTB may also be linked to the idiopathic male infertility that is observed in 30–40% of men with abnormal semen parameters [38,39]. Taken together, this information and the findings of the current investigation imply that there is a direct connection between ZIP9/androgen signaling and TJ protein expression and TJ formation and establish that ZIP9 plays a significant role in male fertility.

Findings from an earlier publication that are based on experiments involving selective ablation of AR in mouse Sertoli cells might indirectly support the conclusions of the current investigation. In this SCARKO animal model, ablation of AR expression affected Sertoli cell maturation, barrier formation, and cytoskeletal development [40]. Nevertheless, the barrier formation was affected only to some degree and in fact was delayed by 5–10 days compared with that of control animals, and ZO-1 expression was only slightly affected. These findings suggest that the classical AR is not the actual promoter of TJ formation. The small effects on TJ formation reported in that work may be associated with the negative influence that AR ablation had on the maturation of the Sertoli cells. Based on their findings, the authors came to the conclusion that the targeted ablation of AR did not completely prevent the formation of an anatomical and functional barrier defining basal and adluminal compartments within the seminiferous epithelium [40]. At that time, however, ZIP9 had not yet been defined as a physiologically relevant androgen receptor of mammalian Sertoli cells.

Testosterone is undoubtedly a major parameter of male physiology. Not only is it essential for the establishment and maintenance of the male phenotype, behavior, and fertility, it also protects male fertility by regulating TJ dynamics at the blood–testis barrier (BTB). Nevertheless, testosterone replacement therapy that is prescribed for low endogenous testosterone levels is not necessarily the best way to treat BTB defects in order to restore fertility. Not only can exogenous testosterone induce severe side effects such as polycythemia, cardiac hypertrophy, and myocardial infarction, it also unfavorably affects male fertility [41,42]. In a hypogonadal mouse model, although administration of androgens triggered TJ formation and also initiated in some tubules the production of post-meiotic elongated spermatids, sperm cells were not obtained [11]. This is because exogenous testosterone, by inducing negative feedback on the hypothalamic–pituitary–gonadal axis, prevents GnRH release from the hypothalamus and FSH and LH from the pituitary, leading to the impairment of testosterone production in Leydig cells [43]. As a result, germ cell production under these conditions is halted [44]. 

Based on the results of the investigation presented here, stimulation of the expression of TJ-associated or TJ-forming proteins by the ZIP9-specific, androgenic peptide IAPG might help to segregate BTB-stimulating events from the negative effects of testosterone on spermatogenesis. Although the expression patterns for TJ proteins appear to be stage and species specific [45], there is some overlap between species. Thus, in testes of Sox8-mutant mice several genes are up- or downregulated. Among them, several that are important for the expression of BTB-associated proteins, such as CLDN3 or CLDN23, are downregulated [46,47]. The importance of claudins, ZO-1, and occludin for the maintenance of the BTB has also been demonstrated in humans. Thus, a recent study dealing with the integrity of the blood–testis barrier in terms of adhesion molecules in nonobstructive azoospermia identified the above proteins as critical parameters not only for BTB formation but also male fertility [48].

Based on the above, one can expect that the selective targeting of ZIP9 by the androgenic peptide IAPG may prove advantageous in the treatment of BTB-associated male infertility. In a first step in that direction, the hypogonadal mouse [49] could be an appropriate model to investigate this possibility.

## Data Availability

Not applicable.

## Supplementary Materials

The following are available online at https://www.mdpi.com/article/10.3390/biology11050668/s1, Figure S1: Original Western blots used in Figure 5; Supplementary Table S1: Antibodies used in the investigation and their providers; Supplementary Table S2: List of primer sequences used for qRT-PCR.
