# Supplementary material for: The Role of ZIP9 and Androgen Receptor in the Establishment of Tight Junctions between Adult Rat Sertoli Cells
Source: Biology (Basel). 2022 Apr 26;11(5):668. doi: 10.3390/biology11050668 (PMC9138102; doi:10.3390/biology11050668)
Supplement: Supplementary file 1 [file biology-11-00668-s001.zip › biology-1645309-supplementary.pdf]

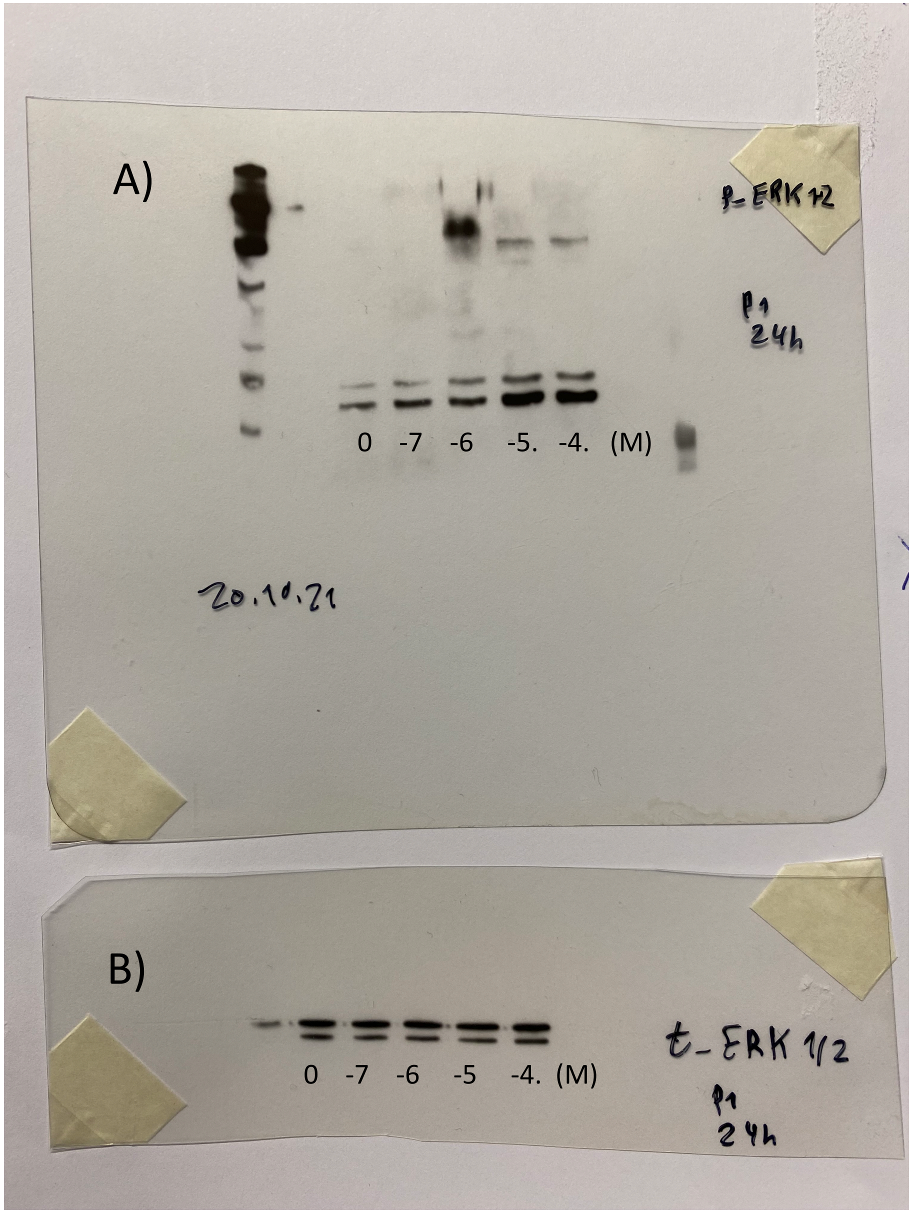

Figure S1: **A)** activation of Erk1/2 phosphorylation by the IAPG peptide. **B)** Total Erk1/2

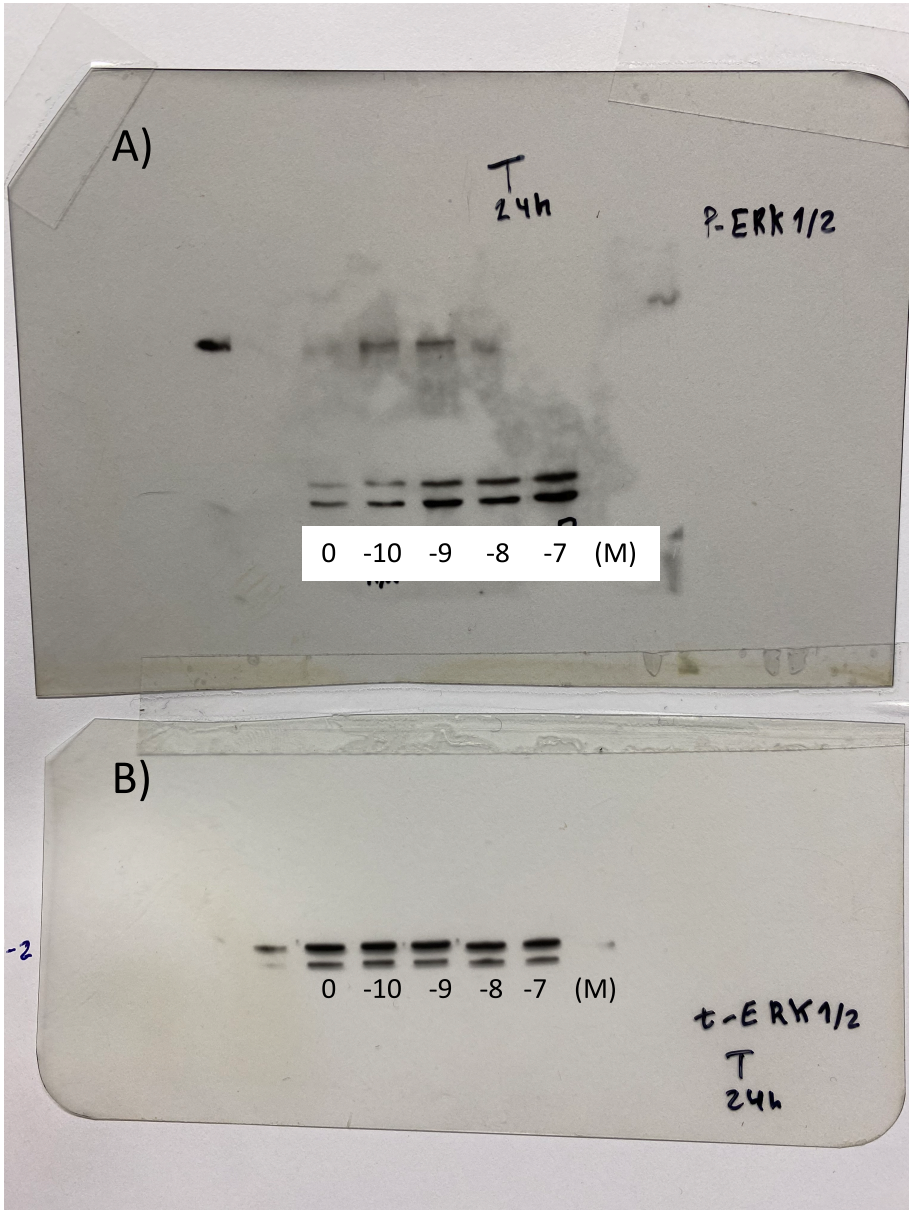

Figure S2: **A)** activation of Erk1/2 phosphorylation by testosterone. **B)** Total Erk1/2
